# Texture evolution during processing and post-processing of maraging steel fabricated by laser powder bed fusion

**DOI:** 10.1038/s41598-022-09977-1

**Published:** 2022-04-16

**Authors:** Rangasayee Kannan, Peeyush Nandwana

**Affiliations:** 1 Manufacturing Science Division, Oak Ridge National Laboratory, Oak Ridge, TN USA; 2 Materials Science and Technology Division, Oak Ridge National Laboratory, Oak Ridge, TN USA

**Keywords:** Engineering, Materials science

## Abstract

In this study, the evolution of solidification texture during LPBF of Ti-free grade 300 maraging steel, and its effect on texture development during subsequent post-fabrication heat treatments was characterized using Electron Backscatter Diffraction (EBSD). It was found that in the as-fabricated state, no texture was observed in the room temperature martensitic phase. However, the reconstructed parent austenite phase displayed a Cube texture with a minor fraction of Rotated Goss texture. During subsequent aging treatments involving two different routes, namely direct aging of the as-fabricated samples, and conventional solution treatment + aging of the as-fabricated samples, significant changes in the texture components of parent austenite were observed, whereas no changes in texture were observed in the room temperature martensitic phase. During direct aging, it was found that with an increase in the aging temperature up to 520 °C, the texture components of the parent austenite changed from Cube/Rotated Goss to Brass, whereas during the conventional solution treatment and aging cycle, interestingly a change in texture component to rotated copper was observed. The transitions in texture components have been discussed using the concepts of recrystallization and twinning in austenite during annealing and/or aging, and strain energy release maximization (SERM) theory. Furthermore, the importance of these preferred orientations on the mechanical properties was quantified using transformation potential diagrams.

## Introduction

Maraging steels are a class of low carbon ultra-high-strength precipitation hardened steels, which obtain their strength from aging of the soft Fe–Ni lath martensitic structure formed after quenching^1^. Due to their superior strength with reasonable ductility, wrought maraging steels have been used extensively in the aerospace industry for the rocket motor casings, hydrospace industry in pressure hulls of submarines, tooling industry, and even for structural applications^1^. Apart from precipitation of strengthening intermetallics during aging, austenite reversion is an important phase transformation that takes place in maraging steels and has been studied in detail by several researchers^2–4^. It is hypothesized that reverted austenite aids in improving the ductility of the part by increasing the elongation at failure by transformation-induced plasticity (TRIP) effect^2–5^, which results in stress/strain-induced transformation of austenite into either ε martensite or α' martensite during plastic deformation. While adequate literature is available on the metallurgical phase transformations in maraging steels, relatively little work has been done on the crystallographic texture evolution in these steels, which is crucial for understanding the anisotropy in mechanical properties as well as understanding the kinetics of the TRIP effect^6,7^. A recent study by Figueiredo et al.^8^ reported a preferred{111} texture along with the forging and rolling direction in martensite during processing, further ascertaining the findings by Hosoya et al*.*^9^ in cold-rolled maraging steels. Abreu et al*.*^10^ found that the as-quenched martensite, upon aging, results in the formation of reverted austenite, and the reverted austenite inherits the texture of parent austenite, which is mainly the {001} component of texture.

Considering their low carbon content and superior weldability, maraging steels are suitable for additive manufacturing. Among the several available additive manufacturing methods, laser powder bed fusion (LPBF) is the most widely used for manufacturing grade 300 maraging steels with several studies available in the literature^11–18^. Most of the literature is on optimizing the process parameters, and microstructure/mechanical properties of the LPBF fabricated parts.

In the case of additively manufactured maraging steels, the evolution of texture during fabrication and post-fabrication heat treatments has not been characterized in detail. In the literature, the texture of room temperature martensite has been reported to be weak due to the rotation in scan strategy between the successive layers, which alters the heat flux direction^19,20^. In all these studies, the texture of the martensite phase has been analyzed and discussed independent of the parent austenite phase which is the first phase to form during solidification. Therefore, crucial information about the development of solidification texture is lost. From the phenomenological theory of martensite crystallography in steels, 24 variants are possible when austenite transforms into martensite^21^. As a result, unless a strong variant selection is observed during martensitic transformation, it is possible for the room temperature martensite to have no texture (random orientation) despite the parent austenite being textured (preferred orientation). Therefore, even though the lack of martensitic texture might not result in anisotropic tensile strength, the textured parent austenite and the subsequently textured reverted austenite formed during aging can influence the deformation-induced phase transformations, and thus, the ductility/toughness of the additively manufactured part. The objective of the present study is to analyze the evolution of solidification texture of parent austenite in Ti-free grade 300 maraging steel manufactured by LPBF during processing and subsequent post-processing heat treatments by reconstructing pole figures, orientation distribution functions from the EBSD data, and understand its influence on the mechanical response of the material.

## Experimental procedure

Ti free grade 300 maraging steel powders with nominal chemical composition as shown in Table 1 were built into cylindrical and cuboidal bars using an Addup FormUp 350 selective laser melting system. The bars were deposited using proprietary process settings, and thus, a range is provided for the laser power (110 W ± 5 W), speed (1500 mm/s ± 50 mm/s), hatch width (50 µm ± 5 µm), and layer thickness (45 µm ± 5 µm).

**Table 1:** Chemical composition of the powders used for fabrication of parts using LPBF.

Element	Ni	Co	Mo	Cr	Al	O	Mn	Si	Ti	N	C	P	S
Wt. %	18.5	9.1	5.0	0.09	0.09	0.08	0.06	0.05	0.04	0.011	0.01	0.006	0.006

Two different aging heat treatments were carried out in a CM Furnaces Inc. Rapid Temp tube furnace following fabrication. Firstly, the as-built samples were aged directly at four different aging temperatures (400 °C, 440 °C, 480 °C, and 520 °C) and two aging times (3 h and 6 h) for each aging temperature. Secondly, the as-fabricated samples underwent conventional solutionizing and aging cycle involving solutionizing at 820 °C (above A temperature) for 1 h followed by aging at 480 °C for 3 h. After aging, the samples were air-cooled to room temperature.

For microstructure characterization, as-fabricated and aged samples were prepared both along and normal to the build direction using conventional mechanical polishing techniques. To understand the evolution of texture and preferred orientation, electron Backscatter Diffraction (EBSD), was carried out using a Zeiss Crossbeam 550 Focused Ion Beam (FIB)/Field Emission Scanning Electron Microscope (FESEM) equipped with an Oxford Symmetry EBSD detector. EBSD measurements were conducted at an accelerating voltage of 25 kV, probe current of 5 nA, and step sizes in the range 0.1–0.3 μm. EBSD data were post-processed using AZtecICE, Channel5, and MTEX/MATLAB post-processing software. The fraction of recrystallized grains was obtained from the EBSD data using the grain shape factor parameter, where a shape factor less than 2.0 indicates recrystallized grains^22,23^, which provides a reliable measurement of the extent of recrystallization in the microstructure. Prior austenite grains were reconstructed from the raw EBSD data using an iterative orientation relationship (OR) determination (which can be either Kurdjumov Sachs (KS) or Nishiyama Wasserman (NW)) between martensite and parent austenite^24^ and Markov clustering^25,26^ using the algorithm developed by Nyyssonen et al*.*^25^ working in conjunction with MTEX/MATLAB^27^. Following reconstruction, the deviation between reconstructed parent austenite and experimentally determined orientations in martensite were determined to identify the suitable OR for reconstruction^25^. It should be noted that when the reconstructions were performed assuming a NW OR for the current set of data, the deviation was found to be higher compared to Kurdjumov Sachs (KS) OR (Refer Appendix Fig. A1). Therefore, all the parent austenite reconstructions in the current manuscript were performed assuming a KS OR. Crystallographic texture components have been identified by reconstructing pole figures (PF) and orientation distribution functions (ODF) from the EBSD data. It should be noted that though such methods are developed for materials subjected to traditional processes such as rolling, where the three directions can be defined, these methods also have been extensively used to explain texture evolution in AM. For the PF and ODF representation contour maps are shown indicating texture intensity as multiples of random orientation. Therefore, though the color bar limits for the different processing conditions are different, the maximum intensity of each processing condition was used to quantify texture evolution. Such a methodology has been used for studying texture in additively manufactured metals^28–31^. Tensile stress–strain curves present in this study were carried out using a strain rate of 0.005 in/in/min until yield followed by an increased strain rate of 0.05 in./in./min until rupture (as per ASTM E8-16a^32^).

## Results

### Texture evolution in as-fabricated and heat treated conditions

**Figure 1:**
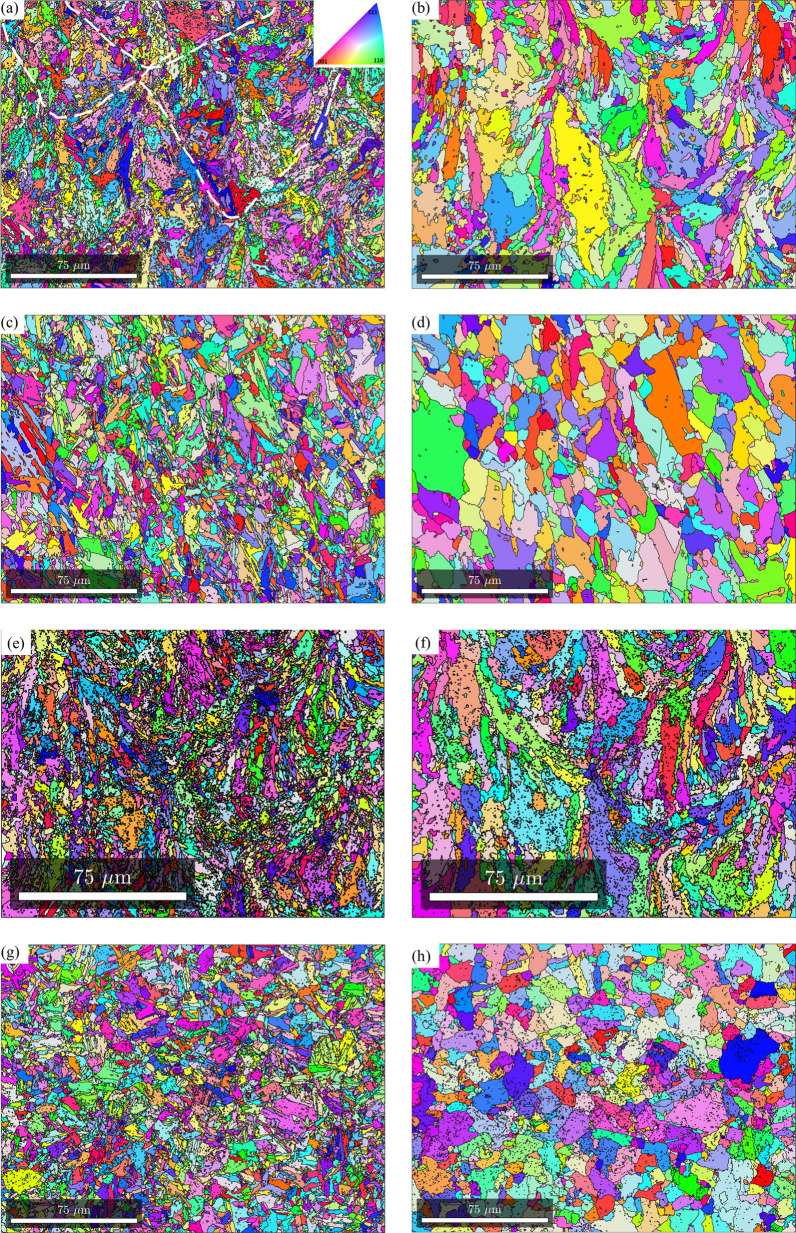
EBSD inverse pole figure maps along the build direction for room temperature martensite (**a**,**c**,**e**,**g**) and reconstructed parent austenite (**b**,**d**,**f**,**h**) for as-fabricated condition (**a**,**b**), solution treated condition (**c**,**d**), direct aged condition (**e**,**f**), and solution treated and aged condition (**g**,**h**).

The typical heat treatment used for maraging steels is a solution treatment followed by aging at 480 °C to achieve peak hardness and strength. Nandwana et al. showed that direct aging at 480 °C results in a higher strength and elongation compared to the conventional two step aging^38^. Therefore, in this section, the results of these different heat treatments on the resulting texture are discussed. Further, an additional sample is considered which was subjected only to solutionizing treatment to serve as a baseline for the texture evolution in solutionzed and aged condition. Figure 1 shows the EBSD inverse pole figure (IPF) map along the build direction (XZ plane). Figure 1a,c,e,g show the room temperature martensite IPF. Melt pool traces can be seen in the as-fabricated and direct aged IPF maps in Fig. 1a,e. Corresponding melt pool traces are also visible in the parent austenite IPF reconstructed from the room temperature martensite in Fig. 1b,f. Upon solutionizing and solutionizing + aging, morphological features indicating the melt pool are no longer visible as shown in Fig. 1c,g for martensite, and corresponding parent austenite grains in Fig. 1d,h. It should also be noted that in the as-fabricated and direct aged conditions, both the martensite and parent austenite grains are elongated along the solidification direction, whereas in the solution treated and solution treated and aged conditions, the grains are equiaxed.

**Figure 2:**
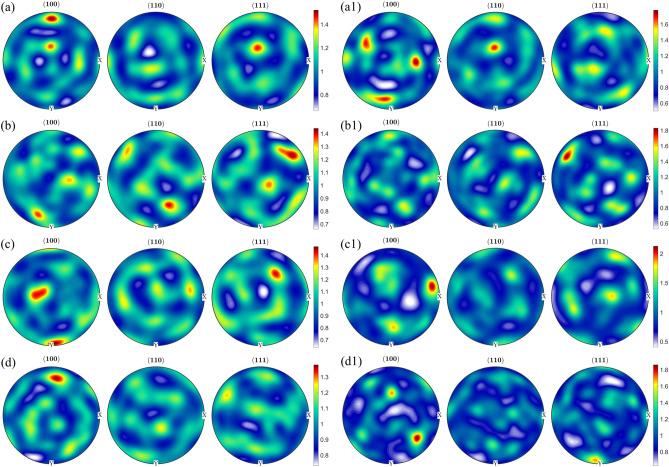
{100}, {110}, and {111} pole figure reconstructions along the build direction for room temperature martensite (**a**–**d**) and parent austenite (**a1**–**d1**) for as-fabricated condition (**a**,**a1**), solution treated condition (**b**,**b1**), direct aged condition (**c**,**c1**), and solution treated and aged condition (**d**,**d1**).

**Figure 3:**
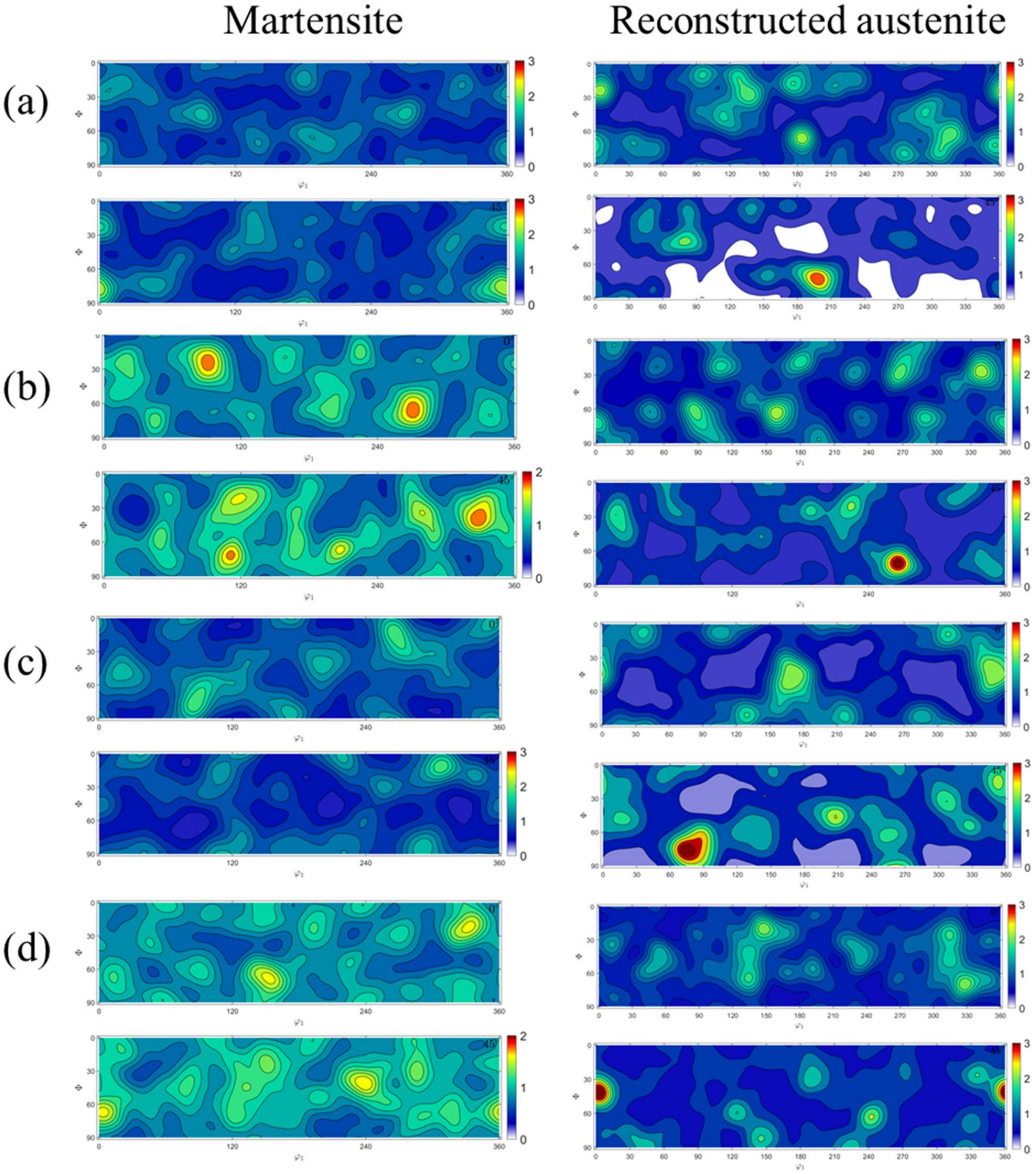
φ_2 _= 45° Orientation distribution function reconstructions along the build direction for reconstructed parent austenite and room temperature martensite for as-fabricated condition (**a**), solution treated condition (**b**), direct aged condition (**c**), and solution treated and aged condition (**d**). The Y-axis is Φ from 0–90 and X-axis if φ_1 _from 0–360.

With regards to the preferred crystallographic orientation, visually it appears that in the as-fabricated and direct aged conditions, significant fraction of grains are oriented along 〈111〉/〈100〉 directions (blue and red colors in the IPF map), whereas in the solution treated and solution treated and aged condition, significant fraction of grains are oriented along the 〈110〉 direction. Figure 2 shows the (100), (110), and (111) pole figures of room temperature martensite and reconstructed parent austenite corresponding to the EBSD maps in Fig. 1. It can be seen that the parent austenite has a relatively stronger texture compared to the room temperature martensite phase. Among the different processing conditions, it can be seen that direct aging at 480 °C results in a higher texture intensity in comparison to the other processing conditions. To quantify the exact orientation ((hkl){uvw}), 0° and 45° φ_2_ section orientation distribution functions (ODF) were reconstrctured using the orientation data for both martensite and parent austenite. The ODF sections confirm that the room temperature martensite has a relatively weaker texture intensity with lack of preferred orientation compared to parent austenite. Comparing the ODF section of austenite to conventional Bunge notation for texture^33^, it can be concluded that in the as-fabricated condition (Fig. 3a), austenite has a preferred cube component ({001}〈100〉) of texture with minor components of rotated goss ({011}〈011〉). Upon direct aging the as-fabricated samples (Fig. 3c), the texture components change towards a preferred near A type/brass type orientation ({110}〈112〉) with minor components of rotated copper (({112}〈011〉)). Interestingly, upon solutionizing and solutionizing and aging (Fig. 3b,d), a strengthening of rotated copper texture is observed. It should be noted that the texture components along the build direction and perpendicular to the build direction are similar (PF, IPF, and ODF’s perpendicular to the build direction are not included here for the sake of brevity of the manuscript), which is likely due to the scan strategy employed. Similar behavior was also observed by other researchers in Refs.^34–37^. Further understanding regarding texture formation as a function of scan strategy requires detailed investigation which is beyond the scope of the current work.

Based on our earlier works, direct aging resulted in better combination of strength and ductility (Refer^38^), and therefore in the next section, only the effect of direct aging temperature on the texture evolution is characterized.

### Effect of direct aging temperatures on texture evolution

**Figure 4:**
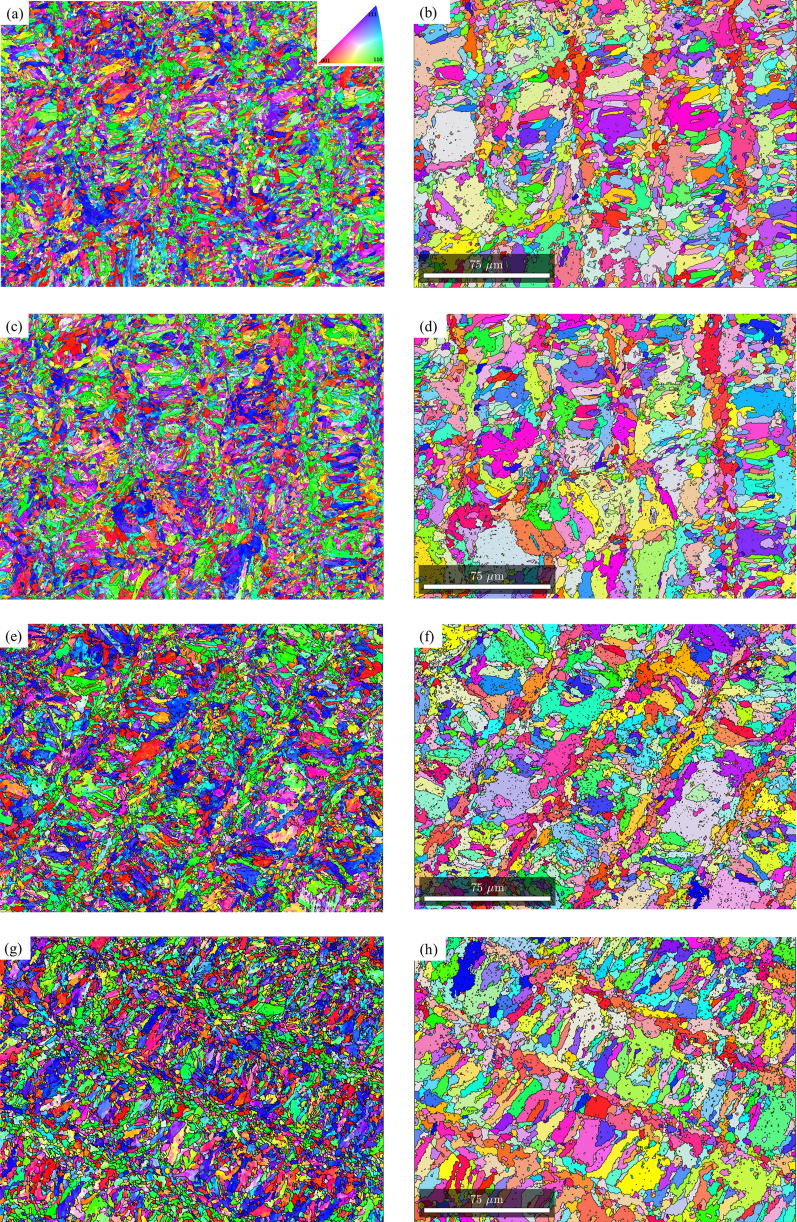

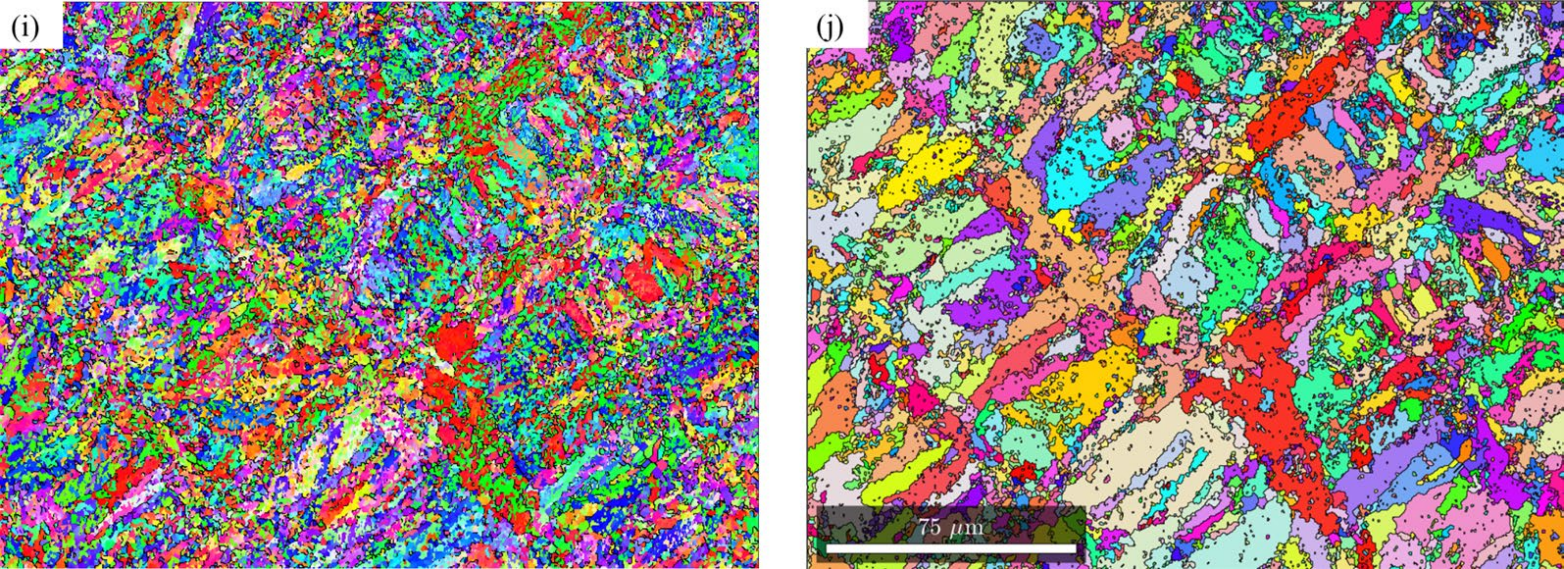
EBSD inverse pole figure maps perpendicular to the build direction for room temperature martensite (**a**,**c**,**e**,**g**,**i**) and reconstructed parent austenite (**b**,**d**,**f**,**h**,**j**) for as-fabricated condition (**a**,**b**), direct aged at 400 °C (**c**,**d**), direct aged at 440 °C (**e**,**f**), direct aged at 480 °C (**g**,**h**), and direct aged at 520 °C (**i**,**j**).

Since the texture components are similar along the build direction and perpendicular to the build direction, for the effect of post-fabrication heat treatments on the texture evolution, all the EBSD analyses were conducted on the sample section perpendicular to the build direction (XY plane). Figure 4 shows the martensite and reconstructed prior austenite IPF’s from the section perpendicular to the build driection for the as-fabricated sample (Fig. 4a,b for martensite and austenite respectively), direct aged at 400 °C (Fig. 4c,d for martensite and austenite respectively), direct aged at 440 °C (Fig. 4e,f for martensite and austenite respectively), direct aged at 480 °C (Fig. 4g,h for martensite and austenite respectively), and direct aged at 520 °C (Fig. 4i,j for martensite and austenite respectively). With regards to the preferred orientation, based on visual inspection, it can be seen that in martensite, majority of the laths are oriented along 〈111〉/〈100〉 direction for as-fabricated and direct aged samples at all temperatures. When the direct aging temperature is 520 °C, very fine equiaxed grains of martensite, indicative of recrystallization, are observed. In the case of parent austenite, until an aging temperature of 480 °C, distinct scan path traces are observed. When the aging temperature is increased to 520 °C, while the scan paths are still visible they are not as distinct as they were at lower tempeatures. Further, very fine austenite grains, indicative of austenite reversion are observed, especially closer to the laser tracks.

**Figure 5:**
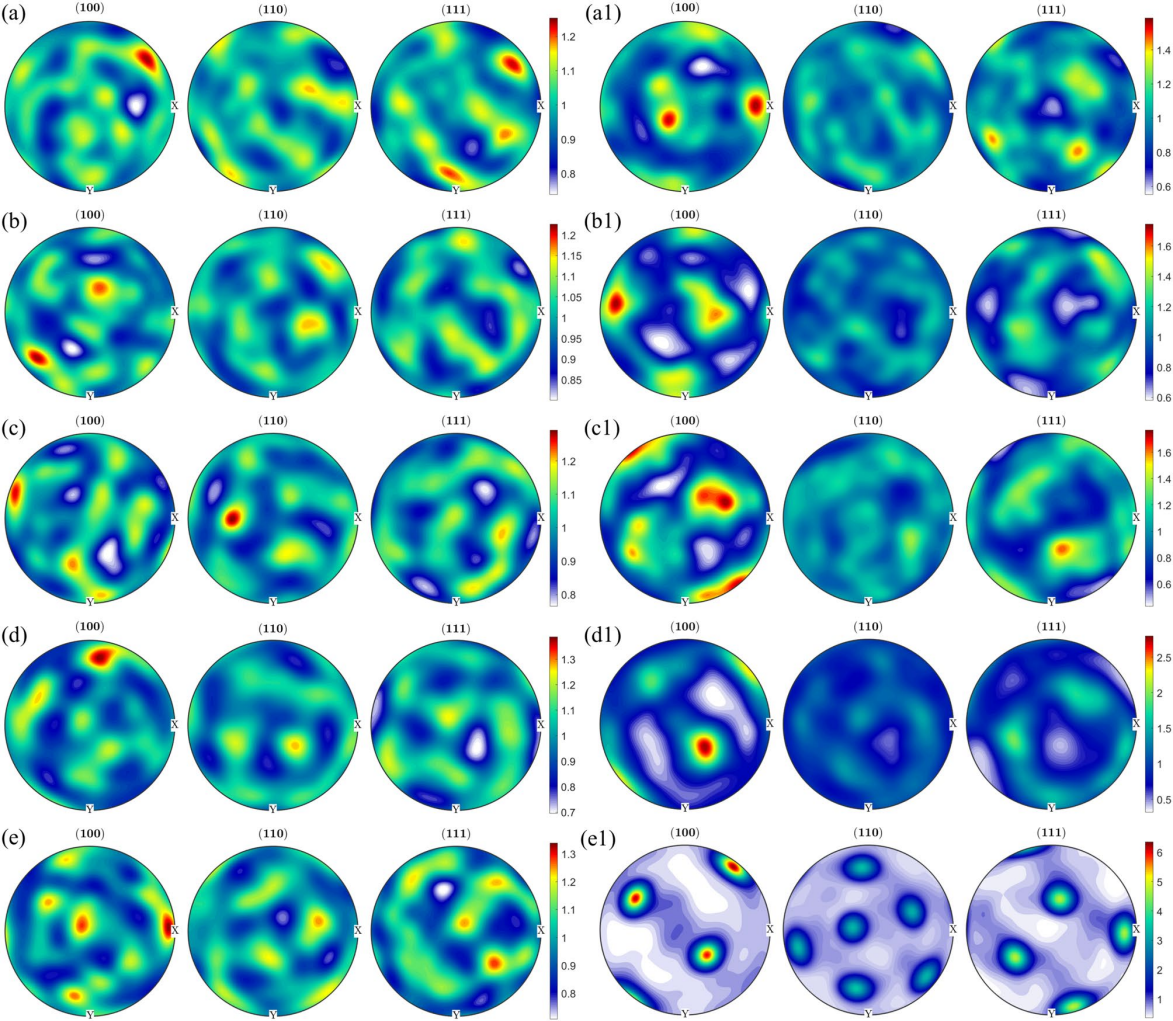
{100}, {110}, and {111} pole figure reconstructions perpendicular to the build direction for room temperature martensite (**a**–**e**) and reconstructed parent austenite (**a1**,**b1**,**c1**, **d1**, and **e1**) for as-fabricated condition (**a**,**a1**), direct aged at 400 °C (**b**,**b1**), direct aged at 440 °C (**c**,**c1**), direct aged at 480 °C (**d**,**d1**), and direct aged at 520 °C (**e**,**e1**).

**Figure 6:**
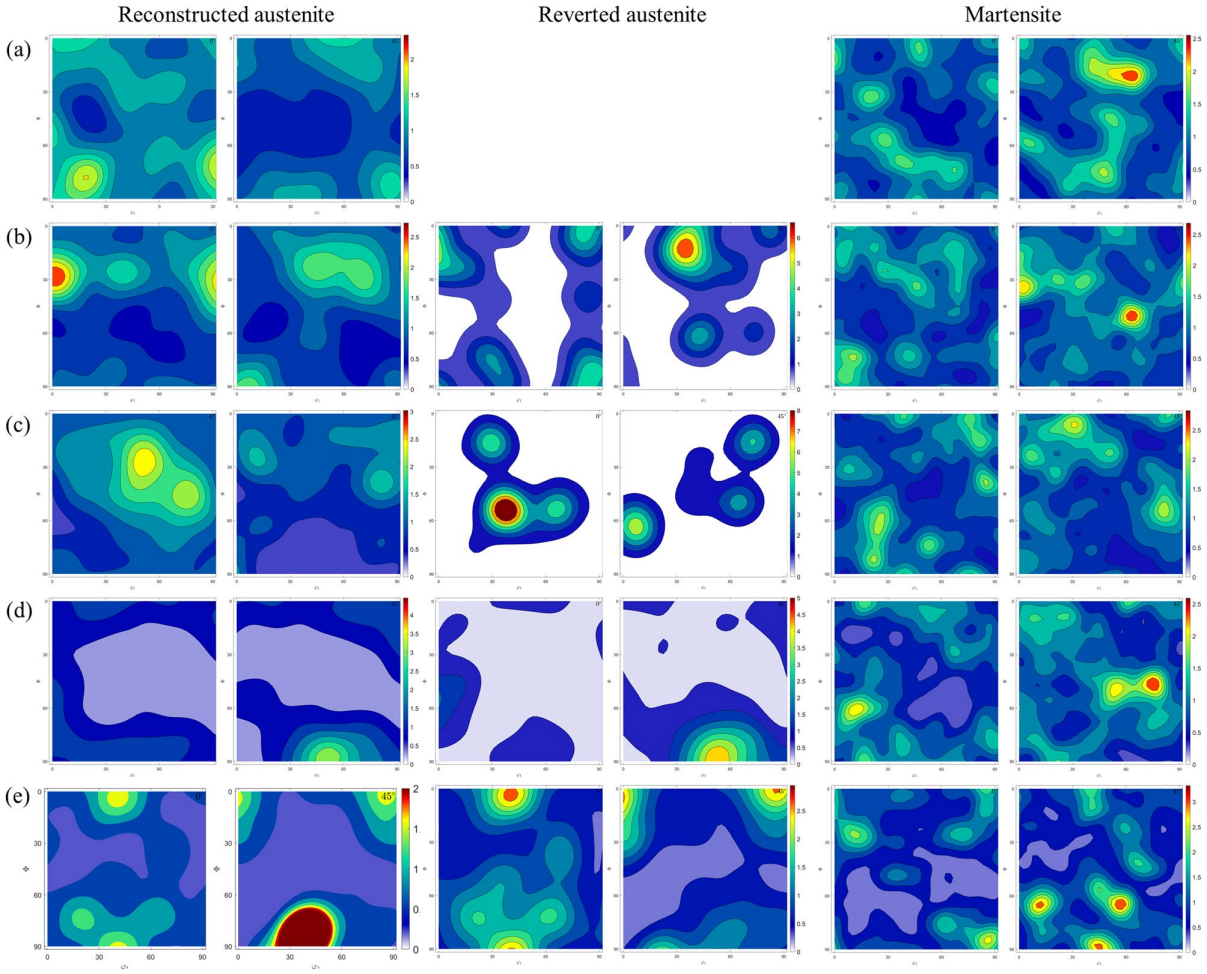
φ_2 _= 45° Orientation distribution function reconstructions perpendicular to the build direction for reconstructed parent austenite, reverted austenite, and martensite for the as-fabricated condition (**a**), direct aged at 400 °C (**b**), direct aged at 440 °C (**c**), direct aged at 480 °C (**d**), and direct aged at 520 °C (**e**). It should be noted that in the as-fabricated condition, retained austenite fraction/size was low enough to be indexed using the step size used for EBSD measurements. The Y-axis is Φ from 0–90 and X-axis if φ1 from 0–360.

To quantify the texture components with varying heat treatments, pole figure and orientation distribution functions were reconstructed for parent austenite and martensite. In the as-fabricated condition, no retained austenite was detected under the EBSD scanning conditions. However, retained austenite was shown to be present in the as-fabircated condition in the inter-lath boundaries of martensite via transmission electron microscopy and could not be resolved by EBSD^38^. On the other hand, as the samples were subjected to direct aging, austenite reversion occurred and the reverted austenite was detected in phase maps (not shown here for brevity). The orientation information for the reverted austenite (which can share the same orientation as the parent austenite) was extracted separately and plotted on the orientation distribution function (ODF) section along with the reconstructed austenite to determine the difference or similarities between the parent austenite and reverted austenite textures upon aging. The results are shown in Figs. 5 and 6. Figure 5 shows the (100), (110), and (111) pole figures of room temperature martensite and reconstructed parent austenite corresponding to the EBSD maps in Fig. 4. It can be seen that martensite has a relatively weaker texture intensity in comparison to parent austenite. Among the different post-processing heat treatment conditions, it can be seen that when the aging temperature is 480 °C and above, a higher texture intensity in the austenite phase is observed compared to the other conditions. Figure 6 shows the 0° and 45° φ_2_ section ODF for parent austenite, reverted austenite, and martensite. The ODF sections confirm that the room temperature martensite has a relatively weaker texture intensity with a lack of preferred orientation compared to parent austenite. It can also be seen from the ODF sections that when the direct aging temperature is 480 °C and higher, a highly preferred austenite orientation is observed.

It is also worth noting that the reverted austenite shares a similar texture component with parent austenite, potentially indicating the growth of parent austenite by dissolution of martensite during direct aging. As discussed above, the retained austenite in as-fabricated condition cannot be reliably detected and indexed by EBSD but since it is the untransformed parent austenite, no distinction has been made between retained and parent austenite. Comparing the ODF sections of austenite to conventional Bunge notation for texture, it can be concluded that in the as-fabricated condition (Fig. 6a), austenite has a preferred cube component ({001}〈100〉) of texture with minor components of rotated goss ({011}〈011〉). Upon direct aging the as-fabricated samples at 400 °C (Fig. 6b), no significant change in the texture components are observed. When the direct aging temperature is increased to 440 °C, strengthening of rotated goss component at the expense of cube component is observed. Additionally, minor fraction of rotated cube component ({001}〈110〉) is observed when the as-fabricated sample is direct aged at 440 °C. When the direct aging temperature is increased to 480 °C, a transition in texture component from rotated goss to near A/brass component ({110}〈112〉) is observed. Finally, increasing the direct aging temperature to 520 °C further strengthening of the near A/brass component of texture is observed.

**Figure 7:**
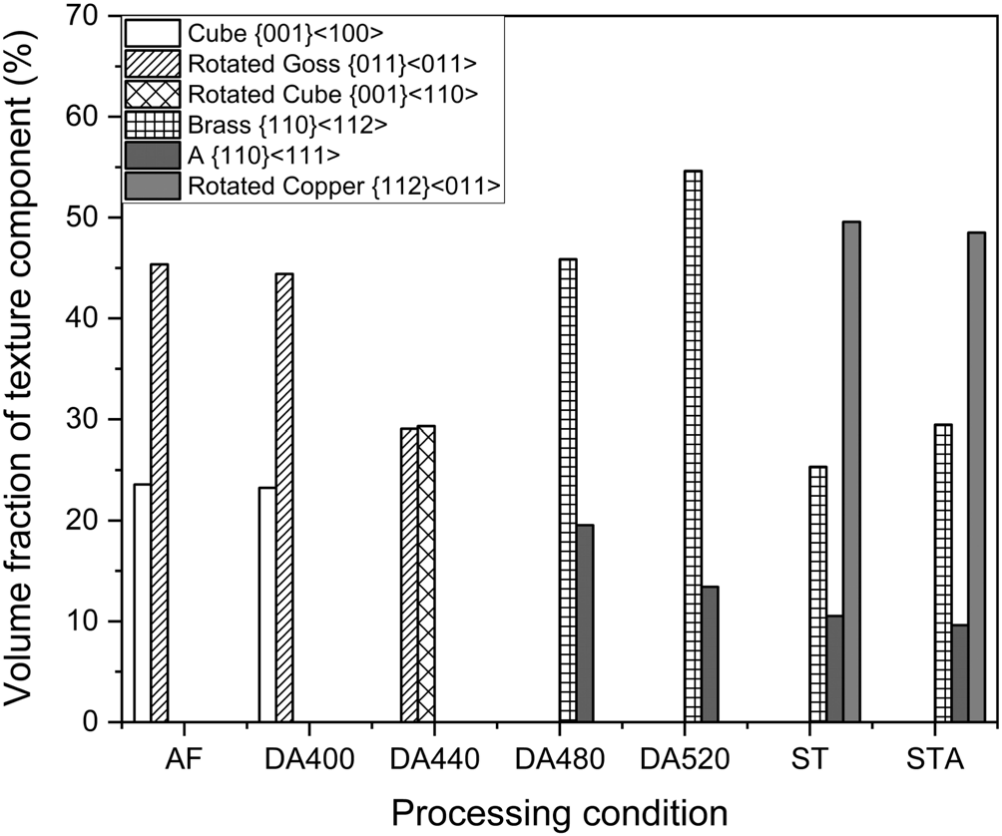
Quantitative volume fraction of texture component evolution under different processing conditions.

Based on the above results (sections “Texture evolution in as-fabricated and heat treated conditions” and “Effect of direct aging temperatures on texture evolution”), we see that different heat treatment strategies such as direct aging, solutionizing, and solutionizing and aging have varied impacts on texture evolution in LPBF Ti-free grade 300 maraging steel. A detailed summary is provided in Table 2 and Fig. 7 to better steer the discussion on these differences in texture evolution as a function of post-processing strategy:

**Table 2:** Summary of texture evolution during various processing conditions in Ti-free maraging steel manufactured using LPBF.

Processing condition	Texture in reconstructed	Texture in retained
	Austenite	Austenite
AF	Cube^a^ {001}〈100〉	
	Rotated Goss^b^ {011}〈011〉	
DA–400 °C	Cube^a^ {001}〈100〉	Cube^a^ {001}〈100〉
	Rotated Goss^b^ {011}〈011〉	Rotated Goss^b^ {011}〈011〉
DA–440 °C	Rotated Goss^a^ {011}〈011〉	Rotated Goss^a^ {011}〈011〉
	Rotated cube^b^ {001}〈110〉	Rotated cube^b^ {001}〈110〉
DA–480 °C	Near A/Brass^a^ {110}〈112〉	Near A/Brass^a^ {110}〈112〉
DA–520 °C	Near A/Brass^a^ {110}〈112〉	Near A/Brass^a^ {110}〈112〉
ST–820 °C	Near A/Brass^a^ {110}〈112〉	Near A/Brass^a^ {110}〈112〉
	Rotated copper^a^ {112}〈011〉	Rotated copper^a^ {112}〈011〉
STA–480 °C	Near A/Brass^a^ {110}〈112〉	Near A/Brass^a^ {110}〈112〉
	Rotated copper^a^ {112}〈011〉	Rotated copper^a^ {112}〈011〉

*AF* As-fabricated, *DA* direct aging post fabrication, *ST* Solutionizing treatment post fabrication, *STA* Aging followed by solutionizing treatment.

^a^Denotes major texture component.

^b^Denotes minor texture component.

## Discussion

### Texture in the as-fabricated condition

**Figure 8:**
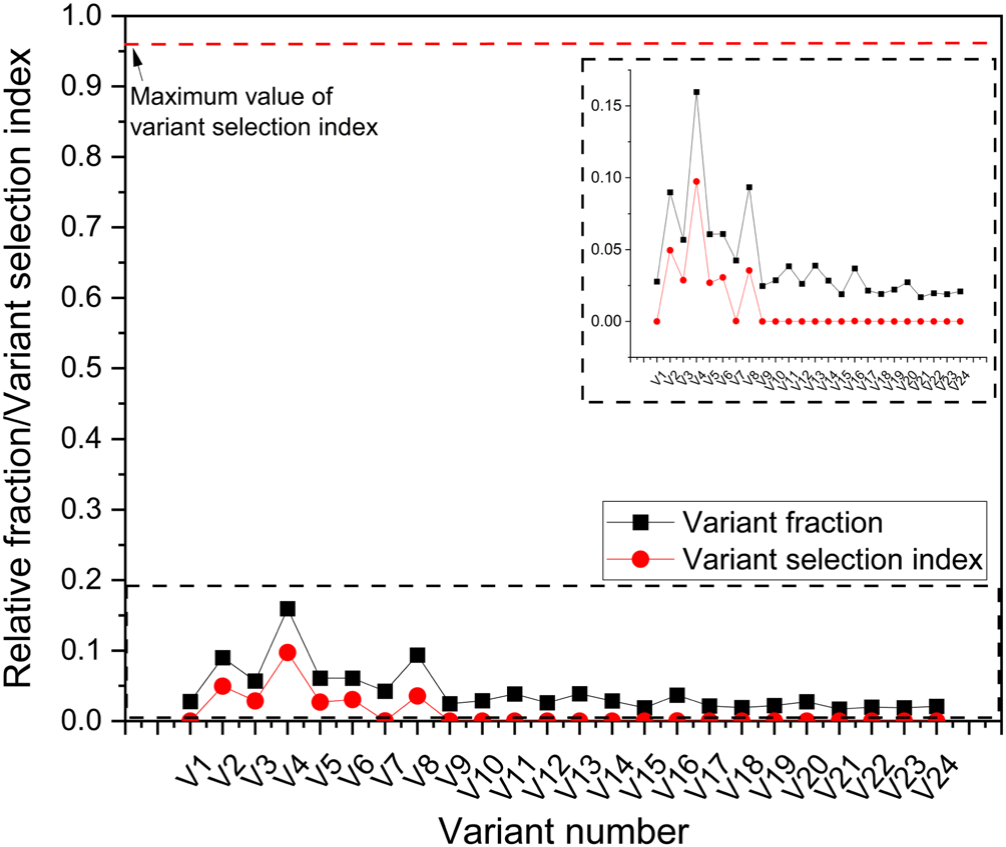
KS orientation relationship variant selection frequency and variant selection index in martensite for the as-fabricated condition.

**Figure 9:**
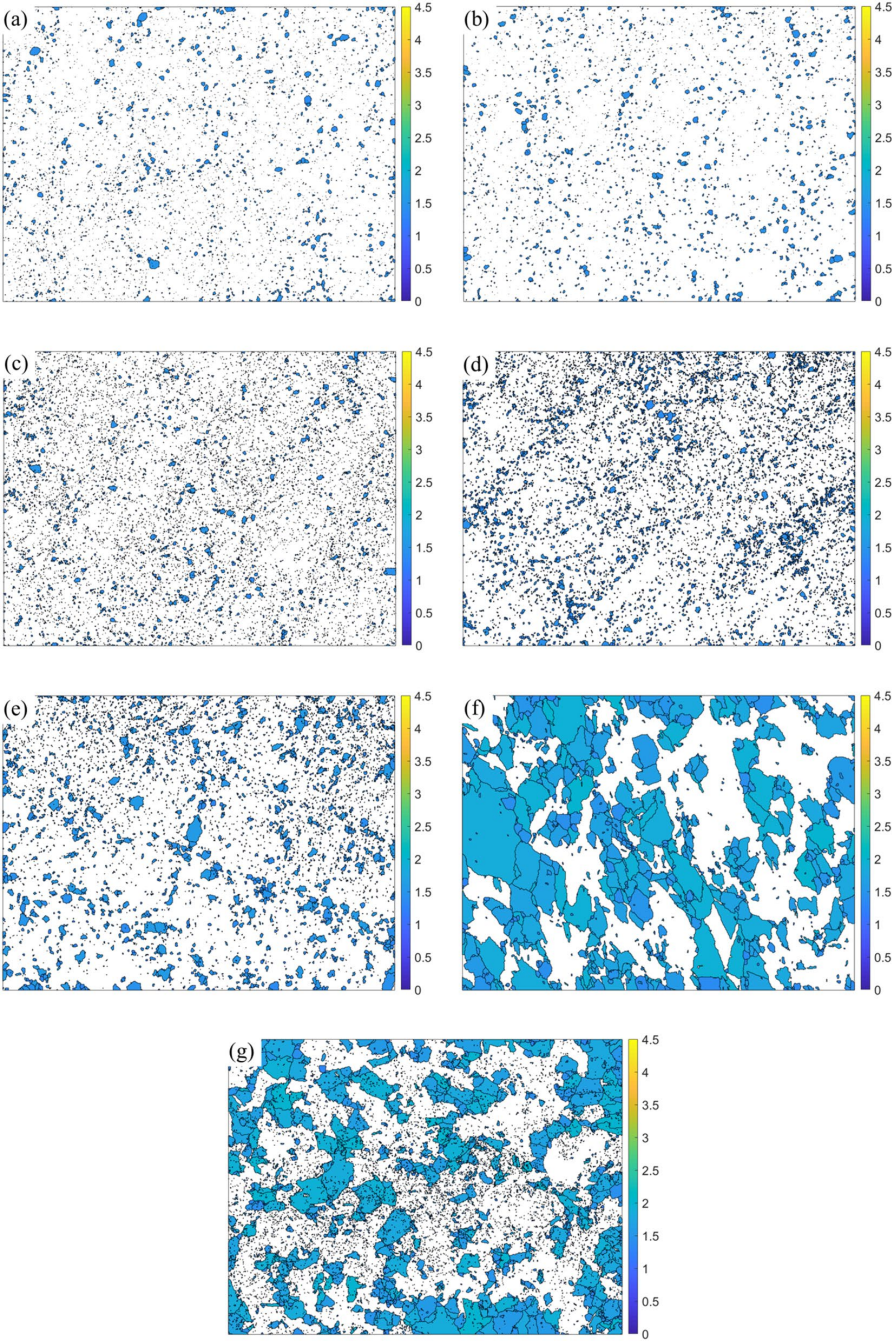
Quantification of fraction of recrystallized austenite grains using grain shape factor. (**a**,**b**,**c**,**d**,**e**,**f**,**g**) indicates the segmented grains with grain shape factor ≤ 2 for the as-fabricated condition, direct aged at 400 °C, direct aged at 440 °C, direct aged at 480 °C, direct aged at 520 °C, solution treated at 820 °C, and solution treated and aged at 480 °C conditions respectively.

Ideally, during additive manufacturing, cubic metals are expected to solidify with a prefferred {001} texture. However, we observe a relatively weaker texture in parent austenite (cube/rotated goss component), the first phase to form during solidifcation, in the as-fabricated condition. The resulting martensite phase during the solid-state phase transformation also exhibits a weaker texture with no preferred variant selection with the formation of all 24 variants, as shown in Fig. 8. The phenomenological theory of austenite–martensite dictates that there is an equal proability of the formation of all 24 variants in the absence of variant selection. Though V4 and V8 appear to have a higher frequency, the average variant selection index (VSI), as proposed by Giri et al.^39^, of V4 and V8 are 0.09 and 0.03 respectively, which is significantly lower considering the maximum value of VSI (0.96) needed to classify specific variants as being preferentially selected. The lack of texture in martensite and weaker texture of austenite in the as-fabricated condition requires further discussion. A lack of texture in martensite fabricated by LPBF based on ex-situ investigations has also been reported by other researchers^19,20^ and was attributed to the variation in direction and magnitude of heat flux due to the rotation of laser scan direction between successive layers. If the change in direction of the heat flux due to rotation of scan direction between successive layers is the only reason for the absence of texture, then there should be no texture in other L-PBF fabricated metals since scan rotation is a common practice in LPBF. However, this is not the case since texture is commonly observed during LPBF of materials such as Inconel 718, Ti-6Al-4 V and stainless steel 316^40–42^. Here we propose three possible hypotheses that might explain the lack of texture in the austenite phase:

- During LPBF, the austenite solidified in the layer n undergoes thermal cycling in the austenite phase field during the deposition of layers n + 1, n + 2, and so on. The repetitive γ − α’ − γ transformations results in generation and progression of misorientations within the γ grain resulting in recrystallization of the initial single crystal γ (with {001} preffered orientation) to poly-crystalline γ with a randomization of texture^43–46^. However, one would expect equiaxed grains resulting from recrystallization. A small volume fraction of equiaxed grains can be seen in Figs. 1b and 4b (later in Fig. 9a) but a complete transformation might be a kinetic limitation to grain growth due to the rapid thermal cycling during fabrication, considering that the recrystallization follows JMAK kinetics with slower rate of recrystallization at lower temperature/time.
- Depending on the process parameters used, the intersection of melt-pool boundaries can result in lack of fusion porosity that might substantially impact the thermal conditions as subsequent layers are deposited. The samples in this work were fabricated using the same process settings used in our earlier work where lack of fusion defects were shown at the intersection of melt pool boundaries^38^. Polonsky et al. showed that during electron beam powder bed fusion of Inconel 718, such lack of fusion defects can result in the formation of stray grains with random textures compared to the bulk of the material^47^. In the as-fabricated condition for LPBF maraging steel, this might result in randomly oriented austenite grains. These randomly orientated austenite grains, upon subsequent cooling will result in the formation of randomly oriented martensite phase with no obvious variant selection.
- Finally, the grain texture and morphology during solidification is strongly dependent on the G–R curves which varies from one alloy to another. The determination of the G–R curve for grade 300 maraging steel is beyond the scope of the study and will be undertaken as a separate study.

Detailed experiements at varying energy density along with computational calculations are required to accurately determine the dominant mechanism for randomization of texture in the austenite phase during solidification, which is beyond the scope of this work. However, once the parent austenite is formed without any dominant texture, the subsequent martensite phase displays a lack of variant selection in line with the phenomenological theory of the austenite to martensite phase transformation.

### Transition in texture from rotated goss component to near A/brass component during post-fabrication aging

Following fabrication, the rotated goss component of texture strengthens at the expense of the cube component of texture during aging. When the aging temperature is increased, the rotated goss component of texture transitions into near A/brass component of texture. It is well known that the Goss component is the preferred recrystallization texture component in austenite^48^. However, the current results show that the texture transitions from rotated goss to near A/brass. Figure 11 shows the recrystallized fraction of austenite grains during aging quantified using the grain shape factor of EBSD^49^. Here a grain shape factor threshold of 2° was used to separate the non-recrystallized grain from the recrystallized grains. It can be seen from Fig. 9 that with the increase in aging temperature, the fraction of recrystallized grains increases (from 9.94% in the as-fabricated condition to 21.3% when the aging temperature is 520 °C).

Thus, it is likely that the transition in texture from rotated goss (when the aging temperature is low) to near A/brass (when the aging temperature is high) is due to the recrystallization of austenite and results in selection of variants which reduce the residual stress in the matrix surrounding a grain^50^. Two of the well reported theories for recrystallization texture include the oriented nucleation theory which states that the preferred activation of a specific nucleus determines the final texture, and the oriented growth theory which states that only the grains which have specific crystallographic relationships with matrix can grow and hence determine the final recrystallization texture^51,52^.

The transition in texture components during recrystallization have been explained using the strain energy release maximization (SERM) theory^48,53,54^. Adopting SERM theory to explain the near A/brass to goss texture component transition during recrystallization, when (110)〈1 $\overline{1 }$ 0〉 are compressed (similar to the residual stresses developed during processing) along [110] direction (near A/brass orientation), the active slip systems can be calculated as (111)[0 $\overline{1 }$ 1] and ($\overline{1 }\overline{1 }$ 1)[101]^45,50,51^. The vector sum of slip direction is [1 $\overline{1 }$ 2] which is the absolute maximum stress direction. Therefore, the recrystallized grain will have texture components along (hk0)[1 $\overline{1 }$ 2], with minimum atomic shuffling (110)[1 $\overline{1 }$ 2] (one of the brass components), and eventually transform into (hk0)[001] which explains the transition from rotated goss to near A/brass. In the current study, during the direct aging and solution treatment conditions, the recrystallization is incomplete and only near A/brass instead of Goss is observed. This further supports our hypothesis (a) in section “Texture in the as-fabricated condition” that the recrystallization kinetics are slower even at aging/solutionizing conditions. This further supports our hypothesis that the parent austenite undergoes recrystallization during LPBF but is kinetically limited due to the rapid thermal cycling since even prolonged aging/solutionizing is not sufficient to complete the recrystallization as evidenced by the lack of complete transformation to Goss component.

### Increase in rotated copper texture component after solutionizing and solutionizing + aging

**Figure 10:**
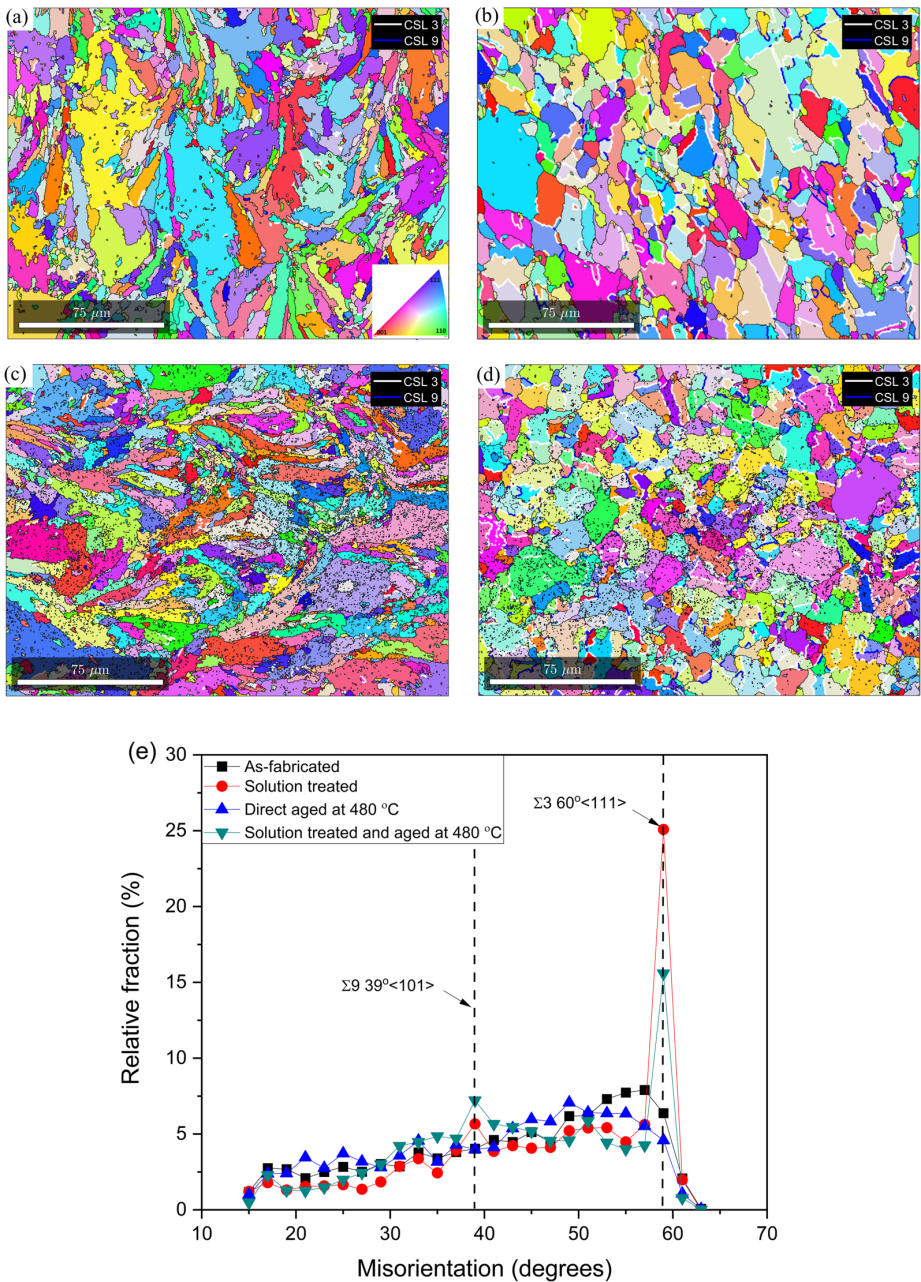
CSL grain boundary analyses indicating Σ3 (white boundaries) and Σ9 (blue boundaries) boundaries for the as-fabricated condition (**a**), solution treated condition (**b**), direct aged condition (**c**), and solution treated and aged condition (**d**). (**e**) Denotes the statistical analysis on the relative fraction of Σ3 and Σ9 boundaries.

Based on SERM theory, upon further recrystallization the texture component is expected to transition to goss. However, the texture results presented in Figs. 3 and 6 show the the texture component transitions from near A/brass to rotated copper upon increasing the aging temperature to 820 °C and further increasing the extent of recrystallization of austenite (Fig. 6). The transition from near A/brass to rotated copper can be explained by the second order twinning of A component ({110}〈111〉)of texture located near the brass component of texture^55^. To verify the second order twinning, CSL (Conincident Site Lattice) boundary analysis was conducted on the as-fabricated sample, which was direct aged at 480 °C, solution treated sample, and the solution treated and aged sample. Σ3 CSL boundary indicative of first order twinning, and Σ9 CSL boundary indicative of second order twinning were characterized. The maximum tolerance of the misorientation angle from the exact axis-angle relationship was identified following the Palumbo-Aust criterion^56^ (i.e. △θ ≤ 15), yielding tolerance limits of 6° for Σ3 and 2.4° for Σ9, respectively, which were used for reconstruction of the CSL boundaries. Figure 10 shows the CSL boundary map along with relative fraction of misorientation for the samples.

It can be seen that in the as-fabricated and 480 °C direct aged samples, no significant fraction of CSL boundaries were observed. However, in the solution treated and solution treated and aged samples, higher fraction of both Σ3 (white boundaries) and Σ9 (blue boundaries) were observed indicating twinning in austenite during aging. Figure 10e shows the relative fraction of misorientation of grain boundaries which quantifies the fraction of Σ3 and Σ 9 boundaries. It can be seen that the fraction of both Σ 3 and Σ 9 boundaries increases drastically when the aging temperature is increased from 480 to 820 °C. Note that 820 °C is above the AC_3_ temperature and anneal twins are quite common in austenite phase at these temperatures. When the solution treated sample is re-aged at 480 °C, the fraction of Σ 3 boundaries decreases however the fraction of Σ 9 boundaries increases.

Therefore, second order twinning can be confirmed based on the CSL boundary analysis, and the presence of this second order twinning resulted in a transition in texture from near brass to rotated copper as hypothesized above. It was proposed by Peters^57^ that the first nuclei for twins form by polygonization with orientations close to the deformed matrix. Subsequently, first and second order twinning take place, with the second order twinning event dominating the developed annealing texture. It should be noted that the generation of first order twins (Σ3) is necessary for the formation of second order twins (Σ9) since the Σ9 boundaries are formed by the twinning reaction Σ3 + Σ3 ⟶ Σ9^58^. This explains the reduction in Σ3 first order twin, and the corresponding increase in Σ9 twins, thereby eventually strengthening the rotated copper component of texture in solution treated and aged sample compared to the sample subjected to solutionizing only.

### Effect of parent austenite texture on mechanical response

**Figure 11:**
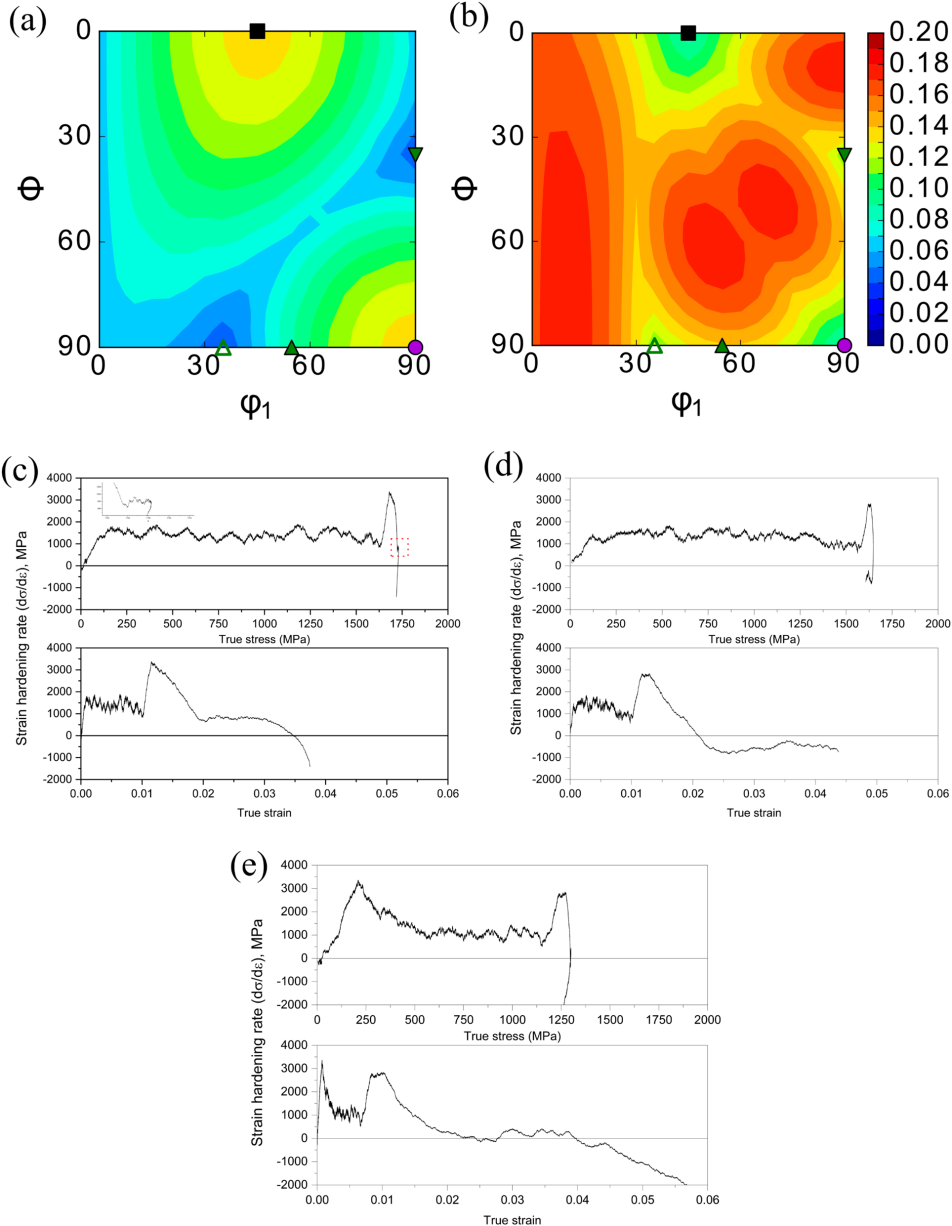
Transformation potential diagrams along the = 45 for and transformation under uniaxial tension (adapted from Ref.^50^) • denotes goss component, ▪ indicates cube component, ▾ indicates Cu component, △ indicates A component, ▴ indicates brass component of texture. (**c**–**e**) Strain hardening analysis indicating TRIP effect for sample aged at 440 °C, 480 °C, and 520 °C. The stress–strain curves for the different aging conditions can be found in Ref.^61^.

It is clear from the results presented above that due to the lack of variant selection during austenite to martensite transformation, the room temperature microstructure has a relatively weaker texture. It should also be noted that the texture components along the build direction (XZ) and perpendicular to the build direction (XY) are the same indicating an isotropic mechanical response. However, parent austenite shows preferred texture evolution during post-processing heat treatments. Since austenite increases the ductility of maraging steels by transformation induced plasticity effect, the concept of transformation potential introduced by Creuziger et al*.*^6,7,59^ is used to explain the role of preferred orientation of austenite on the mechanical response of the additively manufactured material. According to the transformation potential theory, the transformation potential can be expressed as a value derived from Schmid’s law for plasticity which expresses the extent of a specific set of Euler angle combination in austenite to transform into martensite (α') and epsilon martensite (ε) for a given stress state. The transformation potential expressed in = 45° ODF is shown in Fig. 11a,b for transformation of austenite into α' and ε respectively under uniaxial tension. Comparing the transformation potentials to the experimental ODF’s presented in Figs. 3, and 6 it can be concluded that direct aging at 480 °C and 520 °C which have a near A/brass component of texture will result in a lower potential for reverted austenite to transform into martensite (both α' and ε), and hence will have a lower extent of TRIP effect. The existence of TRIP effect in the samples, and the orientation maps of reverted austenite and transformation induced martensite indicating Shoji-Nishiyama OR have been published in our earlier article in Ref.^60^. This is further confirmed by experimental measurements in Fig. 11c–e. Thus, it is clear that apart from the fraction of austenite in the parts, the parent austenite texture from which reverted austenite inherits its texture plays a role on governing the TRIP effect in maraging steel, and current results show that aging at 440 °C provide the best combination of reverted austenite fraction, reverted austenite orientation, strength, and ductility.

## Conclusions

In this study, the evolution of texture in Ti-free variant of grade 300 maraging steel manufactured using laser powder bed fusion during fabrication and post-fabrication heat treatments was characterized using electron backscatter diffraction (EBSD). The following conclusions can be drawn:

1. No preferred orientation was observed in the room temperature martensitic phase in as-fabricated or post-fabrication heat treated conditions. On the other hand, the reconstructed parent austenite showed no preferred orientation in as-fabricated condition but displayed strong textures in the various heat treated conditions. During direct aging, the texture intensity as well as the fraction of recrystallized austenite increased with increasing aging temperature.
2. Upon reconstructing the parent austenite, it was found that in the as-fabricated state, the major preferred orientation components were Cube with minor fractions of Rotated-Goss. During subsequent aging treatments involving two routes, it was found that with the increase in aging temperature up to 520 °C during direct aging, the predominant orientation components changes from Cube/Rotated Goss towards near A/Brass component, whereas during the conventional solution treatment and aging cycle, interestingly an enhancement of rotated-copper type orientations was observed.
3. The transition in texture components from Cube/Rotated Goss to near A/brass can be explained by the recrystallization of reverted austenite during aging, and the enhancement of rotated-copper type orientation during solutionizing can be explained by the second order twinning of A component of texture located in reverted austenite due to an increase in aging temperature.
4. The presence of various texture components in parent austenite affects the texture of reverted austenite likely due to the growth of retained austenite during aging, which in turn affects the transformation potential of austenite to ε martensite or α' martensite during deformation/extent of TRIP effect, thereby reducing the ductility.

The results show that in systems involving solid-state phase transformations post solidification, the solidification texture information can often be overlooked just by observation of orientations of the room temperature microstructure, and highlights the need for development of techniques to provide in-situ orientation information during LPBF.

## Supplementary Information

Supplementary Information.
