# Supplementary material for: Texture evolution during processing and post-processing of maraging steel fabricated by laser powder bed fusion
Source: Sci Rep. 2022 Apr 16;12:6396. doi: 10.1038/s41598-022-09977-1 (PMC9013396; doi:10.1038/s41598-022-09977-1)
Supplement: Supplementary file 1 — Supplementary Information. [file 41598_2022_9977_MOESM1_ESM.docx]

Suuplementary material

Figure A1 shows the deviation between reconstructued austenite and measured martensite orientation assuming both KS and NW OR. Note that NW has a higher deviation from the ideal OR. Therefore, in the manuscript KS OR is considered for reconstruction of parent austenite.


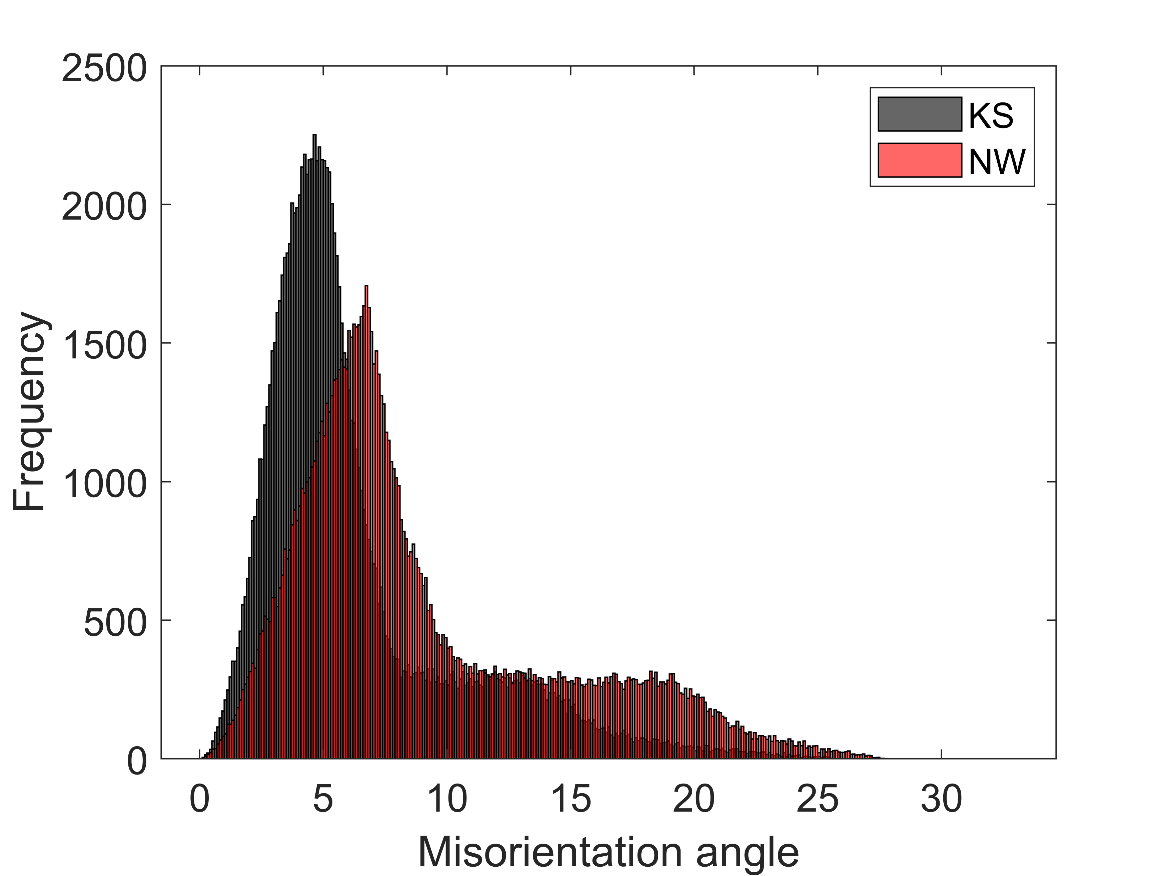


Figure A1: Deviation between reconstructured austenite and measured martensite orientation assuming KS and NW OR.
